# Evaluation of Cardiac Circadian Rhythm Deconditioning Induced by 5-to-60 Days of Head-Down Bed Rest

**DOI:** 10.3389/fphys.2020.612188

**Published:** 2021-01-13

**Authors:** Sarah Solbiati, Alba Martin-Yebra, Pierre Vaïda, Enrico G. Caiani

**Affiliations:** ^1^Department of Electronics, Information and Bioengineering, Politecnico di Milano, Milan, Italy; ^2^Institute of Electronics, Computer and Telecommunication Engineering, Consiglio Nazionale delle Ricerche, Milan, Italy; ^3^Centro de Investigación Biomédica en Red – Bioingeniería, Biomateriales y Nanomedicina, BSICoS Group, Universidad de Zaragoza, Zaragoza, Spain; ^4^College of Health Sciences, University of Bordeaux, Bordeaux, France

**Keywords:** head-down bed rest, heart rate, ventricular repolarization, circadian rhythm, cardiac deconditioning

## Abstract

Head-down tilt (HDT) bed rest elicits changes in cardiac circadian rhythms, generating possible adverse health outcomes such as increased arrhythmic risk. Our aim was to study the impact of HDT duration on the circadian rhythms of heart beat (RR) and ventricular repolarization (QTend) duration intervals from 24-h Holter ECG recordings acquired in 63 subjects during six different HDT bed rest campaigns of different duration (two 5-day, two 21-day, and two 60-day). Circadian rhythms of RR and QTend intervals series were evaluated by Cosinor analysis, resulting in a value of midline (MESOR), oscillation amplitude (OA) and acrophase (φ). In addition, the QTc (with Bazett correction) was computed, and day-time, night-time, maximum and minimum RR, QTend and QTc intervals were calculated. Statistical analysis was conducted, comparing: (1) the effects at 5 (HDT5), 21 (HDT21) and 58 (HDT58) days of HDT with baseline (PRE); (2) trends in recovery period at post-HDT epochs (R) in 5-day, 21-day, and 60-day HDT separately vs. PRE; (3) differences at R + 0 due to bed rest duration; (4) changes between the last HDT acquisition and the respective R + 0 in 5-day, 21-day, and 60-day HDT. During HDT, major changes were observed at HDT5, with increased RR and QTend intervals’ MESOR, mostly related to day-time lengthening and increased minima, while the QTc shortened. Afterward, a progressive trend toward baseline values was observed with HDT progression. Additionally, the φ anticipated, and the OA was reduced during HDT, decreasing system’s ability to react to incoming stimuli. Consequently, the restoration of the orthostatic position elicited the shortening of RR and QTend intervals together with QTc prolongation, notwithstanding the period spent in HDT. However, the magnitude of post-HDT changes, as well as the difference between the last HDT day and R + 0, showed a trend to increase with increasing HDT duration, and 5/7 days were not sufficient for recovering after 60-day HDT. Additionally, the φ postponed and the OA significantly increased at R + 0 compared to PRE after 5-day and 60-day HDT, possibly increasing the arrhythmic risk. These results provide evidence that continuous monitoring of astronauts’ circadian rhythms, and further investigations on possible measures for counteracting the observed modifications, will be key for future missions including long periods of weightlessness and gravity transitions, for preserving astronauts’ health and mission success.

## Introduction

Long duration spaceflights present a number of physiological and psychological stressors that deeply challenge astronauts’ health and performance in space. From the Latin words “circa diem,” meaning “approximately a day,” circadian rhythms are non-random fluctuations having a period of approximately 24 h found in almost every physiological processes (Richards and Gumz, 2013). These rhythms are autonomous, and thus persisting in absence of external synchronizers, but they also entrain to environmental and social cues. In particular, together with the light-dark cycle, a number of factors contribute in maintaining a correct entrainment of circadian rhythms on Earth, such as working and feeding hours, wake/sleep and activity/rest cycles, social interaction, ambient temperature (McKenna et al., 2017), as well as gravity (Fuller et al., 1994). During spaceflight, these conditions are removed: the light-dark cycle is altered, the pull of gravity is reduced, and the regular alternation between standing and supine position is eliminated. A reduction in the strength of the synchronizers directly impacts the characteristics of the circadian rhythms, in terms of altered midline, amplitude and phase, leading to a reduced capacity of adaptation to incoming environmental fluctuations (McKenna et al., 2017). Additionally, circadian rhythm impairment has been correlated on Earth to long-term health problems, including sleep disorders, obesity, diabetes, depression, seasonal affective disorder and aging (Logan and McClung, 2019), with the severity of the consequences increasing with the level of circadian disruption (Gazendam et al., 2013).

Alertness and performance also exhibit circadian rhythmicity (Johnson et al., 1992): as studied in pilots (Caldwell, 2005) and shift workers (Boivin and Boudreau, 2014), when rhythms desynchronization occurs, an increase in the level of fatigue is observed, consequently impairing performance and accentuating the risk of accidents (Flynn-Evans et al., 2016). Episodes of sleep loss, fatigue, and circadian rhythms disruption have been observed in astronauts during past space missions (Manzey and Lorenz, 1998; Monk et al., 1998; Casler and Cook, 1999; Dijk et al., 2001; Flynn-Evans et al., 2016), and have been related to the intense work schedule as well as to environmental factors, such as microgravity, confinement and motion sickness (Guo et al., 2014). Since the maintenance of high level health status and alertness are crucial for the success of a mission, the “*Risk of Performance Decrements and Adverse Health Outcomes Resulting from Sleep Loss, Circadian Desynchronization, and Work Overload*” has also been included in the most recent NASA Human Research Program Integrated Research Plan (NASA Human Research Program, 2020), thus highlighting the need for identifying possible consequences due to circadian desynchronization for individual health and investigating measures for mitigating this risk, particularly in the scenarios of deep space exploration and planetary operations.

Due to the limited possibilities of in-flight research, ground-based analogs are used for reproducing and studying the effects of microgravity on the human body. For example, limb casting and limb suspension have been used to study bone density reduction and neuromuscular alterations due to inactivity and unloading (Kitahara et al., 2003; De Boer et al., 2007; Sanseverino et al., 2018), while bed rest and dry immersion are the most widely used ground analogs for simulating the effects of prolonged weightlessness exposure on the different physiological systems. In particular, Head-Down (−6°) Tilt (HDT) bed rest elicits extensively reduced motor activity (Sandler and Vernikos, 1986), as well as the elimination of the regular alternation between 1 and 0 Gz along the head-to-foot axis, and the characteristic fluids redistribution occurring during sustained exposure to microgravity (Montgomery, 1993). The further neutralization of axial loading and body support is achieved by dry immersion protocols (Navasiolava et al., 2011; Watenpaugh, 2016; Tomilovskaya et al., 2019), which involve immersing the test subject, covered with a highly elastic waterproof cloth, in a tank of thermoneutral water (Shulzhenko et al., 1980; Tomilovskaya et al., 2019). Despite similarities in the effects induced by HDT bed rest and dry immersion (Watenpaugh, 2016; Tomilovskaya et al., 2019), the magnitude of changes on cardiovascular, postural and neuromuscular systems induced by dry immersion resulted up to seven times larger than with HDT bed rest (Tomilovskaya et al., 2018). However, due to its convenience and ease of use (Watenpaugh, 2016), also allowing easier access to countermeasures tested on the subjects, the HDT protocol became the preferred and most utilized ground-based analog of microgravity.

As evidenced in the NASA Human Research Program Integrated Research Plan, there is currently a lack of circadian phase biomarkers easily collectible during spaceflight. Accordingly, HDT bed rest can be used to this aim, by investigating circadian entrainment.

In previous studies during HDT bed rest, we showed that cardiovascular deconditioning is possibly affecting cardiac electrical activity by increasing ventricular repolarization heterogeneity, and thus the risk of inducing rhythm disorders (Caiani et al., 2016). In the same pooled group of subjects examined in this paper, we also showed that, when the normal gravity field is restored, T-wave alternans indices increased after 60-day HDT (but not for shorter durations), indicative of incipient electrical instability on ventricular repolarization (Martín-Yebra et al., 2019).

In addition, our preliminary results on 12 subjects undergoing a 60-day HDT bed rest showed that also the circadian rhythms of both beat-by-beat duration (RR interval) and ventricular repolarization duration (QTend interval) were affected, with a reduction in day/night differences already after 5 days of HDT (Solbiati et al., 2020). As in mice the deficiency or excess of Krüppel-like factor 15, with a role in the transcriptional control of the rhythmic genes expression required for generating the transient outward potassium current, has been related to loss of rhythmic QT variation, abnormal repolarization and enhanced susceptibility to ventricular arrhythmias (Jeyaraj et al., 2012), the importance of further studying in humans the circadianity of RR and QT duration during HDT bed rest as biomarkers appears evident.

Accordingly, our aim was to investigate the changes in the circadian rhythms of cardiac electrical activity from 24-h Holter ECG recordings acquired during six HDT bed rest studies of different duration in a large pooled group of normal subjects, thus expanding the previous results in Solbiati et al. (2020), by evaluating the degree of circadian desynchronization along the HDT, as well as focusing on the changes elicited at HDT discontinuation, as a consequence of the restoration of the gravity field.

This paper is structured as follows: in section “Materials and Methods” the study design and population, data acquisition and processing, as well as the methods utilized for the analysis of circadian rhythms and statistical analyses are described. In section “Results” the results are presented, organized into three sub-sections: (1) focusing on the effects during HDT, considering its different duration; (2) examining the post-HDT bed rest recovery dynamics HDT, separately for the different HDT durations; (3) comparing the effects of gravity field restoration observed after 5-day, 21-day, and 60-day HDT. In section “Discussion” the discussion of the observed changes in the cardiac circadian rhythm is presented, following the same structure adopted in section “Results,” together with a description of the study limitations, and finally in section “Conclusion” the main conclusions are reported.

## Materials and Methods

The data utilized in this study consisted of 12-lead Holter ECG recordings previously acquired in several HDT bed rest campaigns to which our research group participated during the last 10 years. Details on the study design, population, and ECG acquisition protocol will be described in the following sub-sections.

### Study Design and Population

In the context of the European Space Agency head-down bed rest strategy, an only male healthy population was recruited for two short-duration (5 days), two mid-duration (21 days), and two long-duration (60 days) HDT bed rest studies, performed at the Institut de Médecine et de Physiologie Spatiales (MEDES) in Toulouse (France), or at the German Aerospace Center (Deutsches Zentrum für Luft- und Raumfahrt e.V, DLR) in Cologne (Germany). All subjects were not taking medication of any kind.

Volunteers were randomly assigned to a control (CTRL) or to a countermeasure (CM, depending on the study, different interventions were applied to the subjects during HDT to test effectiveness in preventing changes) group. Each study included a period of baseline data collection (PRE), a period of strict 6° HDT bed rest 24 h a day, and a period of post-bed rest recovery, whereas lying in bed during the day was not allowed before and after the HDT period. For detailed description of the design relevant to each campaign, please refer to the Supplementary Material.

The present study considered only the ECG data acquired from the subjects assigned to CTRL groups, for a total of 63 subjects. Sleeping hours were scheduled from 11:00 pm to 6:30 am at DLR and from 11:00 pm to 7:00 am at MEDES, and napping was not allowed during the day. All the performed procedures were in accordance with the 1964 Helsinki Declaration and its later amendments or comparable ethical standards. For each bed rest campaign, all volunteers provided written informed consent to participate in the study, approved by the respective Ethical Committee for Human Research at each of the hosting institutions.

Anthropometric information relevant to the subjects enrolled as CTRL in the 5-day, 21-day, and 60-day HDT campaigns are reported in Table 1, and presented as median [25th percentile; 75th percentile].

**TABLE 1:** Total number of male subjects enrolled in the control group for 5-day, 21-day, and 60-day HDT campaigns, together with their age and Body Mass Index (BMI) expressed as median [25th percentile; 75th percentile].

	**5-day HDT**	**21-day HDT**	**60-day HDT**	***p*-value**

**Number of male subjects**	**22**	**20**	**21**	
Age	31.6[25.6;35.8]	32[28.8;40.3]	28[25;36]	0.4268
BMI	24.4[23.2;25.1]	22.9[21.8;24.5]	24.4[22.5;25.1]	0.14

*The results (*p*-value) of the Kruskal–Wallis test are also presented, with no significant differences between the groups.*

### ECG Data Acquisition and Pre-processing

**FIGURE 1:**
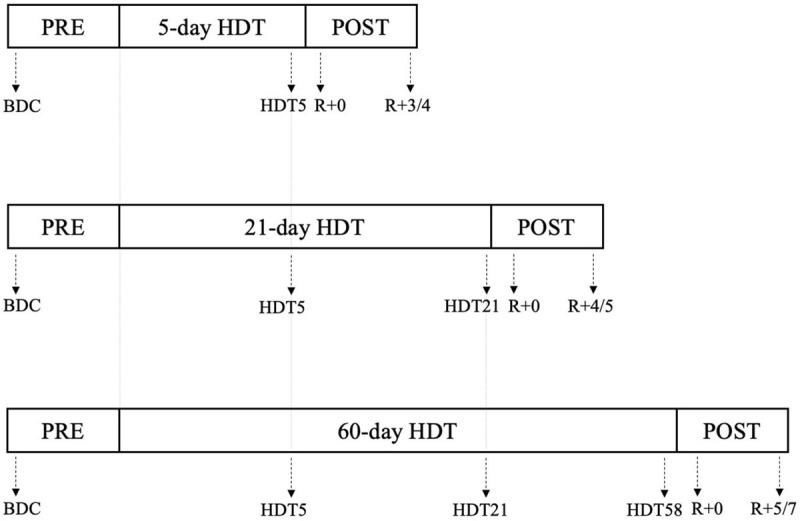
Schematization of the 24 h Holter ECG experiment conducted during the 5-day, 21-day, and 60-day Head-down tilt (HDT) bed rest campaigns. Specific days in which the ECG acquisitions were performed are indicated by the arrows. PRE: baseline data collection (BDC) before the beginning of HDT; HDTx: acquisition performed at day x of the HDT; R + 0: acquisition starting immediately after the conclusion of HDT; R+x: acquisition performed x days after the end of HDT (see section “ECG data acquisition and pre-processing” and Supplementary Materials for details).

For each bed rest campaign, 12-lead 24-h Holter ECGs (1000 Hz, H12+, Mortara Instrument Inc.) were acquired for each subject at specific epochs before, during, and after the HDT period, as schematized in Figure 1. Of note, longer campaigns preserved the same intermediate acquisition days as in shorter campaigns, thus resulting in: 63 subjects from 5-day, 21-day, and 60-day HDT campaigns studied at PRE and HDT5 (i.e., the 5th day of HDT bed rest), of which 41 subjects studied also at HDT21 (i.e., the 21st day of HDT bed rest), and 21 subjects studied also at HDT58 (i.e., the 58th day of HDT bed rest). In addition, all 63 subjects were studied in the 24 h following gravity reinstatement, namely R + 0, as well as several days after, from R + 3 (i.e., the 4th day of recovery) to R + 7 (i.e., the 8th day of recovery), according to the campaign duration, as specified in Figure 1.

For each recording, the fiducial points corresponding to the Q, R, and T wave end (Tend) of the ECG signal were extracted, and beat-to-beat RR and QTend interval series (ms) were computed using the proprietary Mortara Instrument research software SuperECG (Mortara, 2009). The extracted variability series were pre-processed in order to exclude outliers or artifacts due to acquisition problems (i.e., electrode detaching, cable interference, others): an outlier rejection method based on the standard deviation was applied, aimed at eliminating samples exceeding four times the standard deviation of the preceding 50 samples.

Additionally, Bazett’s correction (Bazett, 1920) was applied to compute beat-to-beat QTc interval series, according to the formula:

(1) $$Q\,T\,c\,\left(i\right)=Q\,T\,e\,n\,d\,\left(i\right)/\sqrt{R\,R\,\left(i-1\right)}\;i=1,2,\ldots ,N\,\left(1\right)$$

being N the number of RR and QTend intervals in the series.

Afterward, RR, QTend, and QTc series were realigned to the time of the beginning of the day, and the median values of consecutive, non-overlapping 15-min segments were computed for each subject at each acquisition epoch. Additionally, the minimum and the maximum RR, QTend, and QTc values over the 24 h were computed for each recording, as well as the median value during the diurnal and the nocturnal periods.

### Methods for the Analysis of Circadian Rhythms

The characteristics of circadian rhythm in the cardiac electrical activity were evaluated by performing the single component Cosinor analysis. The Cosinor analysis is a widely used regression method for the evaluation of non-random rhythms in time series, which consists in fitting the original equidistant or non-equidistant series with a periodic function (Cornelissen, 2014), defined by the equation:

(2) Y⁢(t)=M⁢E⁢S⁢O⁢R+O⁢A×cos⁡(2⁢π⁢t/τ+φ)+e⁢(t)

where MESOR (Midline Statistic of Rhythm) represents a rhythm-adjusted mean, OA is the oscillation amplitude, measuring half variation within a night-day cycle, φ is the acrophase, that is the temporal value at which the amplitude of the fitting sinusoid reaches its maximum value, τ is the period representing the duration of one cycle, and e(t) is the fitting error term at each time point, defined as an independent random variable with mean equal to zero and variance σ^2^.

In addition to the Cosinor model, frequency-based approaches such as the Fourier spectral analysis, the Lomb–Scargle periodogram or the autoregressive spectral analysis, allow to perform the spectral decomposition of the series, identifying as circadian those series whose spectrum correlates with a period of 24 h (Leise, 2017). Furthermore, other more specialized methods have been developed in support to the analysis of series characterized by time-variant amplitude or phase, including spectrograms and wavelet transforms (Leise, 2015), as well as empirical (Huang et al., 1998) and non-linear mode decomposition (Iatsenko et al., 2015), in which the original series is decomposed into set of non-linear modes, starting from the time-frequency representation of the series obtained by windowed Fourier transform or wavelet transform.

Unlike the Cosinor analysis, however, the cited methods require at least 3 or preferably more consecutive cycles acquired from the same source or individual. In the present study, multiple, non-consecutive, 24-h-long series were available, and thus the analysis of the changes in the circadian period was not allowed. Accordingly, the circadian period τ was assumed to be equal to 24 h (Cornelissen, 2014), being synchronized to the external 24-h light-dark cycle, which was fixed and imposed by the experimental protocol in all the considered studies. Thus, being τ assumed as known, the equation (2) can be re-written as:

(3) Y⁢(t)=M⁢E⁢S⁢O⁢R+β⁢x+γ⁢z+e⁢(t)

where β = A(cosφ), γ =−A(sinφ), x = cos(2πt/τ), z = sin(2πt/τ). Afterward, the least squares method is used to minimize the residual sum of squares (RSS), i.e. the sum of squared differences between measurements Y(t_*i*_), for i = 1, 2, …, N, and the values estimated from the model (Cornelissen, 2014):

(4) $$R\,S\,S=\sum_{i}{\left[Y_{i}-\overset{\wedge }{M\,E\,S\,O\,R}+\overset{\wedge }{\beta }x_{i}+\overset{\wedge }{\gamma }z_{i}\right]}^{2}$$

Trigonometric formulae are then used to derive estimates of the amplitude (OA) and acrophase (φ) from $\overset{\wedge }{\beta }$ and $\overset{\wedge }{\gamma }$.

Consequently, in the present work, the circadian rhythm of 24-h RR and QTend series was evaluated for each recording by performing the single component Cosinor analysis on the computed 15-min medians, to minimize fitting errors of the least square method in presence of possible outliers, thus resulting in a value of MESOR, OA and φ_*QTend*_ and φ_*RR*_ for each subject at each epoch. Recordings having less than 60% of available signal over the 24 h were excluded from the analysis. In all other cases, linear interpolation was performed to fill missing samples.

Additionally, the difference in minutes between the RR and QTend acrophases was computed as:

(5) $$\Delta \,\varphi =\varphi_{R\,R}-\varphi_{Q\,T\,e\,n\,d}$$

### Statistical Analysis

The performed statistical analyses are described in detail in the following sub-sections. For all tests, *p* < 0.05 was considered as level of significance (α) to reject the null hypothesis.

#### Presence of Circadian Rhythms

The presence of circadian rhythmicity in RR and QTend series was assessed by the Zero-Amplitude test (Cornelissen, 2014), with the null hypothesis that the amplitude of the series is zero, and thus that there is no rhythm in it.

#### Effect of HDT Bed Rest Duration

In order to test the null hypothesis that HDT, independently of its duration, did not produce any effect on the computed parameters, the non-parametric Kruskal–Wallis test for independent samples (due to the different number of subjects in each group for this comparison) was performed to compare values obtained at PRE, HDT5, HDT21, and HDT58, with the *post hoc* Mann–Whitney test with Bonferroni correction.

#### Post-HDT Bed Rest Recovery

To test the null hypothesis that in the same subject HDT did not produce any effect on the computed parameters at its conclusion and in the following days, the non-parametric Friedman test for repeated measures was applied, together with *post hoc* Wilcoxon Signed Rank test with Bonferroni correction, to compare values at PRE, at R + 0 and at the following available R+x epoch (with x depending from the HDT duration – see Figure 1), separately after 5-day, 21-day, and 60-day HDT. In case of missing data, the relevant subject was excluded from the paired comparison.

#### Effects of Gravity Field Restoration

To test the null hypothesis that HDT discontinuation, independently of its duration, did not produce immediate effects on the computed parameters, the difference (expressed as Δ%) between the last available HDT acquisition and the respective R + 0 was computed for 5-day, 21-day, and 60-day HDT campaigns separately, according to the formulae:

(6) $$\Delta^{5-d\,a\,y\,H\,D\,T}=\frac{\left(R+0\right)-\left(H\,D\,T\,5\right)}{H\,D\,T\,5}\times 100$$

(7) $$\Delta^{21-d\,a\,y\,H\,D\,T}=\frac{\left(R+0\right)-\left(H\,D\,T\,21\right)}{H\,D\,T\,21}\times 100$$

(8) $$\Delta^{60-d\,a\,y\,H\,D\,T}=\frac{\left(R+0\right)-\left(H\,D\,T\,58\right)}{H\,D\,T\,58}\times 100$$

Thus, the Kruskal–Wallis test (*p* < 0.05) was performed to compare Δ^5–day HDT^, Δ^21–day HDT^, and Δ^60–day HDT^, together with *post hoc* Mann–Whitney test with Bonferroni correction.

## Results

Results are presented as median [25th percentile; 75th percentile], and detailed information about statistical analyses results are reported in the tables. In the text, only significant variations are underlined (unless otherwise specified) in terms of median of the relative change (in %) compared to respective values at PRE.

Due to acquisition problems, the recordings specified in Table 2 were not available for the analyses. Consequently, the results available from those subjects with missing recordings were excluded from all the analyses involving paired comparisons, while they were still considered in unpaired tests.

**TABLE 2:** List of the number of subjects and corresponding head-down tilt (HDT) bed rest campaign (using its acronym as defined in Supplementary Material) in which day, night or Cosinor analyses were not performed for certain epochs because of missing data.

**Epoch**	**Day**	**Night**	**Cosinor**
PRE		1 SAG	1 SAG, 1 RSL
HDT5		1 MNX	1 MNX, 1 RSL
HDT21		1 MNX	1 MNX
R + 0 5-day HDT	1 BR-AG1	1 BR-AG1	1 BR-AG1
R + 0 21-day HDT	1 MEP	1 MEP	1 MNX, 1 MEP
R + 4/5 21-day HDT	4 MEP	1 MNX, 4 MEP	1 MNX, 4 MEP
R + 0 60-day HDT		1 RSL	1 RSL

*PRE, days of observation before HDT was started; HDTx, acquisition performed at day x of the HDT; R + 0, acquisition starting immediately after the conclusion of HDT; R+x, acquisition performed x days after the end of HDT (see section “ECG data acquisition and pre-processing” for details).*

Locally missing data within the 24 h, reported both as % in respect of the whole acquisition and as median [25th percentile; 75th percentile] durations, were found at PRE (1.04%, 15[0;90] minutes), at HDT58 (2.08%, 30[0;94] minutes), and at R + 0 in 21-day HDT bed rest (2.08%, 30[15;67] minutes).

### Effect of HDT Bed Rest Duration

**FIGURE 2:**
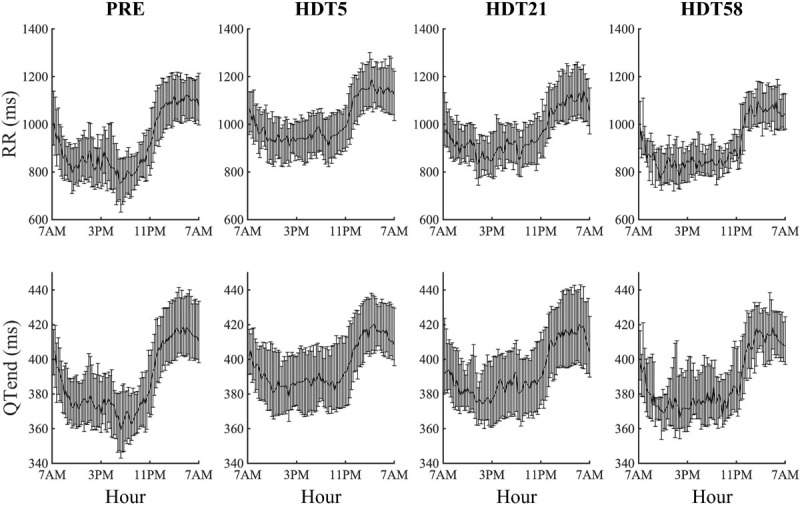
Cumulative results (each value represents the 15-min median with its 25th and 75th percentiles) of heart beat duration (RR) and ventricular repolarization duration (QTend) computed from the corresponding pooled population before (PRE) the beginning of head-down tilt (HDT) and at the x-th day of HDT bed rest (HDTx).

Both at PRE and at each HDT bed rest epoch, RR and QTend interval series exhibited a marked circadian pattern, as visible in Figure 2 in which the distributions over the 24 h (represented as a median [25th-75th] value every 15-min) of the RR and QTend intervals for the pooled population are shown. In particular, as confirmed by the Zero-Amplitude test, the null hypothesis was rejected and the circadian rhythm was maintained in all subjects at both PRE and at all HDT epochs. As expected, shorter RR intervals were visible during the day, denoting higher heart rate, followed by a steep increase toward the higher values observed at night, with QTend interval following RR variations.

However, the characteristics of the circadian rhythm changed during the HDT period. Table 3 provides a summary of day, night, maximum and minimum values of RR, QTend and QTc intervals, as well as of the Cosinor parameters for RR and QTend, at PRE and at each HDT epoch.

**TABLE 3:** Results expressed as median [25th percentile; 75th percentile] of the analysis of the effects of head-down tilt (HDT) duration on the parameters computed from heart rate (RR) and ventricular repolarization duration, as original values (QTend) and corrected using the Bazett formula (QTc), in the pooled population before HDT was started (PRE: 63 subjects day, 62 subjects night, 61 subjects Cosinor) and at each available HDT epoch during the bed rest (HDT5: 63 subjects day, 62 subjects night, 61 subjects Cosinor; HDT21: 41 subjects day, 40 subjects night and Cosinor; HDT58: 21 subjects).

		**PRE**	**HDT5**	**HDT21**	**HDT58**	***p*-value**
RR	Day (ms)	818[763;907]	955[870;1006]*^(7e–08)^	897[820;967]*^(3e–03)^	826[791;908]^✦ (1e–03)^	3.2e-07
	Night (ms)	1089[992;1188]	1140[1055;1209]	1076[1016;1187]	1035[983;1120]^✦ (7e–03)^	0.037
	Maximum (ms)	1187[1090;1291]	1246[1149;1334]	1191[1101;1318]	1150[1068;1234]	0.070
	Minimum (ms)	531[391;650]	740[696;824]*^(2e–14)^	721[628;782]*^(4e–07)^	691[600;770]*^(6e–05)^	3e-14
	MESOR (ms)	915[852;998]	1019[931;1081]*^(4e–05)^	962[917;1037]	903[862;961]^✦ (8e–04)^	6.3e-05
	OA (ms)	160[137;201]	112[90;144]*^(3e–06)^	122[105;160]*^(1e–03)^	120[98;151]*^(1e–03)^	8.3e-06
	φ (hour:min)	3:46[3:15;4:19]	2:14[2:31;4:08]	3:30[3:03;4:09]	3:16[2:43;3:43]*^(6e–03)^	0.032
QTend	Day (ms)	374[365;388]	388[370;403]*^(1e–03)^	381[369;401]	374[366;393]	0.014
	Night (ms)	413[399;432]	411[401;430]	415[395;434]	409[399;427]	0.870
	Maximum (ms)	428[411;451]	424[411;443]	428[405;449]	421[412;440]	0.813
	Minimum (ms)	338[302;350]	365[352;380]*^(1e–10)^	361[348;375]*^(2e–06)^	358[343;368]*^(7e–04)^	3.7e-10
	MESOR (ms)	386[375;401]	395[380;412]	392[379;409]	387[377;402]	0.176
	OA (ms)	25[20;32]	17[13;20]*^(4e–10)^	19[16;23]*^(1e–04),✦(7e–03)^	18[15;23]*^(2.7e–04)^	7.4e-10
	φ (hour:min)	3:58[3:09;4:33]	3:19[2:40;3:52]*^(2e–03)^	3:33[3:10;4:08]	3:27[2:55;3:44]*^(6.3e–03)^	0.004
**Δ**φ	φ (min)	–10[–25.4;3.3]	–2.1[–19.6;12.2]	–5[–18.9;13.9]	–8[–17.3;–1.7]	0.161
QTc	Day (ms)	409[400;422]	399[388;410]*^(1e–04)^	403[395;416]	415[397;424]^✦ (4e–03)^	4.0e-4
	Night (ms)	398[388;411]	387[378;399]*^(3e–03)^	394[382;404]	402[385;412]	0.014
	Maximum (ms)	446[428;462]	420[414;427]*^(1e–10)^	427[417;439]*^(3e–05)^	433[422;443]	5.6e-10
	Minimum (ms)	384[374;400]	377[367;390]	385[371;397]	393[376;408]	0.022

MESOR: midline statistic of rhythm; OA, oscillation amplitude; φ, acrophase. The *p*-value column refers to the result of the Kruskal–Wallis test. Results of the *post hoc* Mann–Whitney test with Bonferroni correction are presented as: ^*(p–value)^: *p* < 0.05/6 vs. PRE; ^✦ (^*^*p*^*^–value)^: *p* < 0.05/6 vs. HDT5.

Major changes were observed at HDT5, with increased RR and QTend MESOR compared to PRE (+11.4% and +2.3%, respectively), mostly related to a lengthening occurring during the diurnal period (RR: +16.8%; QTend: +3.7%), as also evidenced by the increased minima (RR: +39.4%; QTend: +8%). On the other hand, no changes were observed in RR and QTend maxima and in nocturnal values for any of the HDT epochs. With the prolongation of HDT a gradual recovery toward baseline values was observed: for the RR interval, this was manifested at HDT58 as decreased diurnal (−13.5%), nocturnal (−9.2%), and MESOR (−11.4%) values compared to HDT5. However, the RR interval was still lengthened compared to PRE at HDT21 during the day (+9.7%), and both RR and QTend minima remained higher than at PRE up to HDT58, showing only a trend toward the baseline (RR: +35.8% at HDT21, +30.1% at HDT58; QTend: +6.8% at HDT21, +5.9% at HDT58).

The increased minimum and diurnal values, and the simultaneous unchanged maximum and nocturnal values, during the entire HDT caused a dampening in the amplitude of day-night oscillations (OA) for RR (between −23.8 and −30%) and QTend (between −24 and −32%) from baseline as the HDT was prolonged.

The φ_*QTend*_ occurred earlier during the HDT compared to PRE, and a similar trend (significant only at HDT58) was observed in φ_*RR*_.

Also the QTc interval was affected during the HDT, being particularly shortened at HDT5 compared to the baseline (Day −2.4%; Night −2.8%; Maximum −5.8%) reaching values lower than the 390 ms normality limit for short QT syndrome (Rautaharju et al., 2009). Afterward, only maximum QTc remained shortened at HDT21 (−4.3%), while day and night values trended toward recovery, being greater at HDT58 when compared to HDT5.

### Post-HDT Recovery

In this paragraph, the results relevant to the analysis of post-HDT recovery are presented in terms of comparison between the baseline and the recovery epochs, for 5-day, 21-day, and 60-day HDT campaigns separately.

#### Recovery After 5-Day HDT

The results relevant to the 5-day HDT bed rest are reported in Table 4.

**TABLE 4:** Relative changes of the immediate (R + 0) effects of recovery, and following same days (R + 3/4), after a 5-day head-down tilt (HDT) on the parameters computed from heart rate (RR) and ventricular repolarization duration, as original values (QTend) and corrected using the Bazett formula (QTc), compared to baseline values (PRE) as reference.

			**Relative changes compared to PRE**		
	**5-days HDT**	**PRE**	**R + 0**	**R + 3/4**	***p*-value**
RR	Day (ms)	850[770;907]	−8.3[−15.8;−6.6]%*^(7e–05)^	+0.6[−2.8;3.8]%^✦ (6e–05)^	6.0e-07
	Night (ms)	1093[984;1181]	−3.9[−7.6; −1.2]%*^(1e–03)^	+4.5[−0.9;6.9]%^✦ (1.e–04)^	1.1e-05
	Maximum (ms)	1159[1072;1271]	−3.5[−5.0; −0.4]%*^(1e–02)^	+5.1[1.9;9.1]%*^(3e–03), ✦ (1e–04)^	1.9e-05
	Minimum (ms)	554[401;665]	−29.7[−43.2; −3.5]%*^(8e–04)^	+12.8[−8.5;57.7]%^✦ (6e–05)^	4.4e-06
	MESOR (ms)	917[851;1001]	−6.7[−10.3; −4.6]%*^(1e–04)^	+3.5[0.4;6.6]%*^(8e–03), ✦ (9e–05)^	1.6e-07
	OA (ms)	150[108;191]	+28.1[1.2;58.4]%*^(3e–03)^	+4.7[−17.2;26.3]%^✦ (0.01)^	0.022
	φ (hour:min)	h 4:00[3:35;4:47]	0.18[−0.55;0.91] hours	−0.53[−1.52;0.88] hours	0.387
QTend	Day (ms)	376[366;387]	−4.1[−6.5; −1.3]%*^(4e–04)^	+0.5[−1.6;2.3]%^✦ (6e–05)^	1.8e-05
	Night (ms)	409[398;435]	−1.1[−5.0;1.1]%	+1.5[−1.6;3.4]%^✦ (7e–03)^	4.8e-03
	Maximum (ms)	422[408;444]	−0.9[−3.5;1.5]%	+2.[−0.5;4.2]%^✦ (1e–03)^	0.002
	Minimum (ms)	340[279;349]	−9.5[−16.6; −0.1]%*^(4e–03)^	+6.3[−3.5;25.2]%^✦ (6e–05)^	1.0e-05
	MESOR (ms)	385[377;401]	−2.6[−4.6; −1]%*^(4e–03)^	+1.4[−0.2;3.7]%^✦ (1e–04)^	1.1e-05
	OA (ms)	23[18;33]	+26.9[0.2;60.8]%*^(5e–03)^	−11.8[−24.6;20.8]%^✦ (3e–04)^	0.001
	φ (hour:min)	h 4:14[3:56;5:03]	+0.21[−0.35;1.08] hours	−0.72[−1.28;0.42] hours	0.445
**Δ**φ	φ (minutes)	−12.8[−32.7;4.4]	−7.1[−25.4;13.2] minutes	0.3[−22.8;33] minutes	0.350
QTc	Day (ms)	409[399;419]	+2[−0.3;3.7]%	+0.9[−1.0;2.7]%	0.228
	Night (ms)	399[391;411]	+0.6[−1.5;2.2]%	−0.4[−1.4;1.3]%	1
	Maximum (ms)	439[425;453]	+3.6[−1;7.2]%	+1.2[−1;3.4]%	0.100
	Minimum (ms)	383[374;401]	+1.5[−1.3;4.3]%	+0.9[−0.7;2.6]%	0.538

MESOR, midline statistic of rhythm; OA, oscillation amplitude; φ, acrophase. Results are expressed as median [25th percentile; 75th percentile] representing the relative change compared to PRE (in %). The *p*-value column refers to the result of the Friedman test. Results of the *post hoc* Wilcoxon Signed Rank test with Bonferroni correction are presented as: ^*(^*^*p*^*^–value)^: *p* < 0.05/3 vs. PRE; ^✦ (p–value)^: *p* < 0.05/3 vs. HDT5.

At R + 0, the diurnal and nocturnal RR interval decreased with respect to the baseline (Day −8.3%, Night −3.9%), as well as in terms of minimum (−29.7%), maximum (−3.5%), and MESOR (−6.7%) values, with QTend following RR variations (Day −4.1%, Night −1.1%, Minimum −9.5%, MESOR −2.6%). Moreover, the OA of both RR and QTend was increased at R + 0 by +28.1 and +26.9%, respectively. At R + 3/4, a trend toward recovery was observed. In most of the cases, this was visible as a return toward baseline values, that for RR maximum and MESOR was manifested as an increase compared to PRE (+5.1 and +3.5%, respectively). No changes were detected in RR and QTend acrophases and in their difference, as well as for the QTc.

#### Recovery After 21-Day HDT

The results relevant to the 21-day HDT bed rest are reported in Table 5.

**TABLE 5:** Relative changes of the immediate (R + 0) effects of recovery, and following same days (R + 4/5), after a 21-day head-down tilt (HDT) on the parameters computed from heart rate (RR) and ventricular repolarization duration, as original values (QTend) and corrected using the Bazett formula (QTc), compared to baseline values (PRE) as reference.

			**Relative changes compared to PRE**		
	**21-days HDT**	**PRE**	**R + 0**	**R + 4/5**	***p*-value**
RR	Day (ms)	838[778;915]	−11.2[−18.8; −7]%*^(4e–04)^	−5.8[−12.1;0]%^✦ (7e–03)^	2.1e-05
	Night (ms)	1078[1024;1198]	−11.5[−15.5; −3.6]%*^(6e–04)^	−1.5[−9.8;2.6]%^✦ (0.01)^	0.008
	Maximum (ms)	1232[1148;1314]	−8.3[−12; −3.7]%*^(1e–03)^	−2.1[−8.2;0.2]%	0.003
	Minimum (ms)	402[375;416]	−1.5[−6.7;3]%	+23.9[4.0;56.7]%^✦ (5e–03)^	0.015
	MESOR (ms)	927[887;1019]	−11[−13.3; −7.9]%*^(1e–04)^	−3.3[−11.9;0.9]%^✦ (7e–03)^	2.3e-04
	OA (ms)	183[155;209]	−8.5[−37.0;30.8]%	−8.4[−27.6;1.4]%	0.168
	φ (hour:min)	3:52[3:35;4:29]	+0.1[−0.2;1.1] hours	−0.9[−1.1; −0.3] hours*^(2e–03)^, ^✦ (0.01)^	0.005
QTend	Day (ms)	374[367;393]	−2.9[−6.0; −0.3]%*^(3e–03)^	−0.3[−2.5;2.8]%^✦ (4e–03)^	0.003
	Night (ms)	420[411;433]	−1.4[−6.3;0.6]%	+2.5[−0.4;3.7]%^✦ (1e–04)^	0.001
	Maximum (ms)	439[424;558]	−4.1[−11.9;0.1]%	−1.6[−17.6;3]%	0.305
	Minimum (ms)	308[298;339]	−0.8[−7.9;4.6]%	+9.0[−0.7;15.3]%^✦ (2e–03)^	0.003
	MESOR (ms)	392[384;414]	−2.8[−6.2; −1.2]%	−0.6[−1.4;4.2]%^✦ (1e–03)^	2.5e-03
	OA (ms)	28[21;34]	−14.5[−37.7;27.5]%	−3.6[−22.1;18.3]%	0.807
	φ (hour:min)	3:54[2:27;4:24]	+0.90[0.28;2.65] hours	−0.48[−1.09; −0.01] hours	0.010
**Δ**φ	φ (minutes)	−7.3[−15;6.6]	−29.3[−55; −17.5]	−10.9[−27.4;4.4]	0.076
QTc	Day (ms)	408[395;425]	+3.3[1.2;5.3]%*^(6e–03)^	+2.9[0.8;4.7]%*^(2e–03)^	0.001
	Night (ms)	401[383;414]	+2.1[0.9;3.2]%*^(7e–03)^	+3.2[1.5;4.4]%*^(1e–04)^	1.4e-04
	Maximum (ms)	467[459;629]	−0.1[−4.5;2]%	−1.4[−13.7;2.1]%	0.646
	Minimum (ms)	390[370;398]	+2.4[0.2;3.4]%	+4.0[1.5;4.8]%*^(5e–04)^	6.7e-04

MESOR, midline statistic of rhythm; OA, oscillation amplitude; φ, acrophase. Results are expressed as median [25th percentile; 75th percentile] representing the relative change compared to PRE (in %). The *p*-value column refers to the result of the Friedman test. Results of the *post hoc* Wilcoxon Signed Rank test with Bonferroni correction are presented as: ^*(^*^*p*^*^–value)^: *p* < 0.05/3 vs. PRE; ^✦ (^*^*p*^*^–value)^: *p* < 0.05/3 vs. HDT5.

At R + 0, the RR interval appeared shortened compared to PRE, in terms of day (−11.2%), night (−11.5%), maximum (−8.3%), and MESOR (−11%) values. Conversely, the QTend interval resulted shortened only during the day (−2.9), while diurnal and nocturnal QTc lengthened (+3.3 and +2.1%, respectively). Afterward, at R + 4/5, RR and QTend intervals recovery toward baseline values was reached, except for diurnal, nocturnal and minimum QTc that continued to be higher than PRE (+2.9, +3.2, and +4%). The RR acrophase was slightly anticipated compared to PRE while that of QTend was not modified.

#### Recovery After 60-Day HDT

The results relevant to the 60-day HDT bed rest are reported in Table 6.

**TABLE 6:** Relative changes of the immediate (R + 0) effects of recovery, and following same days (R + 5/7), after a 60-day head-down tilt (HDT) on the parameters computed from heart rate (RR) and ventricular repolarization duration, as original values (QTend) and corrected using the Bazett formula (QTc), compared to baseline values (PRE) as reference.

			**Relative changes compared to PRE**		
	**60-days HDT**	**PRE**	**R + 0**	**R + 5/7**	***p*-value**
RR	Day (ms)	796[754;868]	−27.5[−35.7; −20.9]%*^(6e–05)^	−10[−13.6; −6.3]%*^(2e–04), ✦ (6e–05)^	4.6e-09
	Night (ms)	1094[1017;1197]	−22.5[−32.9; −16.1]%*^(2e–04)^	−12.7[−15.8; −8.6]%*^(4e–04), ✦ (2e–03)^	2.2e-07
	Maximum (ms)	1201[1091;1285]	−21[−26.4; −10.9]%*^(6e–05)^	−10.4[−13.7; −4.8]%*^(7e–05), ✦ (6e–05)^	2.0e-09
	Minimum (ms)	624[560;666]	−26.4[−39; −13]%*^(9e–05)^	−13.4[−26.7; −1.6]%*^(6e–03), ✦ (1e–01)^	9.1e-07
	MESOR (ms)	899[861;988]	−24.6[−34.2; −18.3]%*^(1e–04)^	−10.1[−14.6; −8.5]%*^(2e–04), ✦ (1e–04)^	1.4e-08
	OA (ms)	168[145;204]	−2.1[−18.9;21]%	−19.3[−26.9;8.1]%	0.019
	φ (hour:min)	3:23[3:00;3:56]	+0.98[0.3;1.9] hours*^(4e–03)^	−0.04[−0.6;0.99] hours	0.019
QTend	Day (ms)	372[359;386]	−10[−15.1; −7.2]%*^(6e–05)^	−2.4[−4.2;0]%*^(9e–03),✦(6e–05)^	3.4e-08
	Night (ms)	414[340;427]	−7.0[−10.5; −5.7]%*^(1e–03)^	−1.4[−5.9; −0.1]%^✦ (3e–03)^	6.1e-06
	Maximum (ms)	426[410;441]	−6.4[−9.6; −3.8]%*^(1e–03)^	−0.8[−4.7;1.5]%^✦ (4e–03)^	1.8e-04
	Minimum (ms)	342[328;350]	−11.9[−15.6; −8.8]%*^(7e–05)^	−3.5[−13.3;0.7]%^✦ (5e–04)^	1.3e-05
	MESOR (ms)	385[371;400]	−8.3[−13.8; −6.4]%*^(2e–03)^	−2.9[−5.2;0.1]%^✦ (3e–03)^	4.8e-05
	OA (ms)	26[24;31]	+38.4[4.5;49.8]%*^(2e–03)^	+15[−15.6;27.9]%^✦ (9e–03)^	0.021
	φ (hour:min)	3:38[2:58;4:13]	+1[0.15;1.97] hours*^(7e–04)^	−0.36[−0.83;0.47] hours^✦ (1e–03)^	0.001
**Δ**φ	φ (minutes)	−14.1[−24.6; −5.7]	−0.9[−28.6;15.6]	+16.9[−1.1;38]*^(0.01), ✦ (3e–03)^	0.007
QTc	Day (ms)	408[402;423]	+5.3[0.9;8]%*^(2e–04)^	+3.2[0.4;4.8]%*^(6e–03),✦(7e–03)^	4.3e-04
	Night (ms)	398[389;411]	+3.9[1.5;7.4]%*^(3e–04)^	+4.4[1.6;7.3]%*^(2e–03)^	1.2e-04
	Maximum (ms)	438[427;457]	+4.2[1.9;8.1]%*^(2e–04)^	+2.5[0.4;5.3]%*^(9e–3)^	2.2e-05
	Minimum (ms)	383[376;400]	+4.1[1.6;7.4]%*^(3e–04)^	+4.6[0.5;6.6]%*^(3e–03)^	0.002

MESOR, midline statistic of rhythm; OA, oscillation amplitude; φ, acrophase. Results are expressed as median [25t h percentile; 75th percentile] representing the relative change compared to PRE (in %). The *p*-value column refers to the result of the Friedman test. Results of the *post hoc* Wilcoxon Signed Rank test with Bonferroni correction are presented as: ^*(^*^*p*^*^–value)^: *p* < 0.05/3 vs. PRE; ^✦ (^*^*p*^*^–value)^: *p* < 0.05/3 vs. HDT5.

At R + 0, both RR and QTend intervals significantly shortened compared to baseline, in terms of diurnal (RR: −27.5%; QTend: −10%), nocturnal (RR: −22.5%; QTend: −7.0%), maximum (RR: −21%; QTend: −6.4%), minimum (RR: −26.4%; QTend: −11.9%), and MESOR (RR: −24.6%; QTend: −8.3%) values. Conversely, the QTc resulted prolonged at R + 0 with respect to the baseline (Day +5.3%; Night +3.9%; Minimum +4.1%). These changes in RR and QTc still persisted at R + 5/7, showing only a slight trend toward recovery.

While the OA of RR did not show significant variations at R + 0, the OA of QTend resulted increased by +38.4% compared to PRE, and then recovering at R + 5/7.

Both RR and QTend acrophases resulted postponed at R + 0, but recovered at R + 5/7, where a slight anticipation of RR over QT in Δφ was evidenced.

### Effects of Gravity Field Restoration

**FIGURE 3:**
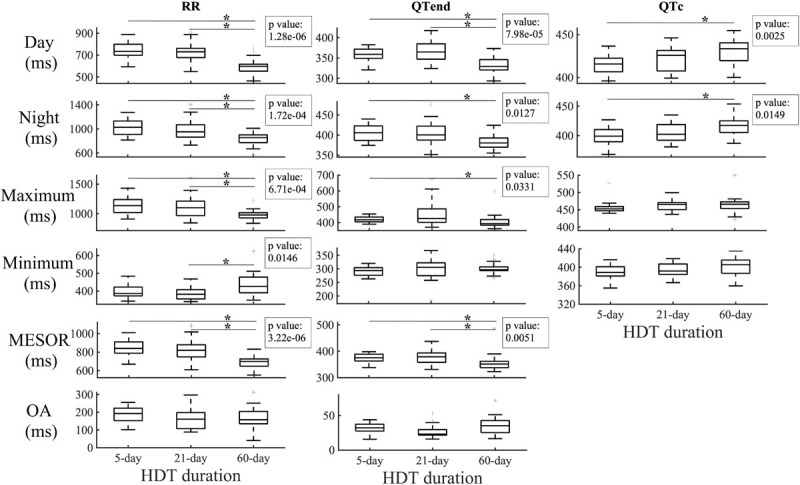
Comparison of the effects of early recovery (R + 0) for the 5-day, 21-day, and 60-day duration head-down tilt (HDT) bed rest campaigns in diurnal, nocturnal, maximum, minimum and Midline Statistic of Rhythm (MESOR) values obtained from the beat-to-beat series of heart beat (RR) and ventricular repolarization duration, expressed as original values (QTend) or corrected using the Bazett formula (QTc). In the textbox, the *p*-value of the Kruskal–Wallis test is indicated. *p < 0.05/3 *post hoc* Mann–Whitney tests with Bonferroni correction.

A comparison of RR, QTend and QTc values among 5-day, 21-day, and 60-day HDT campaigns at the respective R + 0, i.e., immediately after gravity restoration, is reported in Figure 3.

After 60 days of HDT, the RR interval appeared more reduced compared to after 5-day HDT (Day −20.6[−30.1;−10.3]%, Night −16.2[−20.6;−10.3]%, Maximum −13.5[−18.2;−6.4]%, MESOR −17.1[−21.1;−12.8]%) and to after 21-day HDT (Day −18.3[−22.9; −11.7]%, Night −10.2[−27.8; −6.4]%, Maximum −10.4[−25.4; −2.2]%, MESOR −12.9[−23.7; −9.3]%). A similar behavior was observed in QTend day (−7.8[−11.4; −2.3]% compared to 5-day HDT, −7.0[−12.4; −3.8]% compared to 21-day HDT), night (−3.9[−7.9; −0.1]% compared to 5-day HDT), maximum (−3.8[−9.0;1.2]% compared to 5-day HDT), and MESOR (−6.2[−9.3; −0.3]% compared to 5-day HDT, −7.3[−11.3; −0.7]% compared to 21-day HDT) values. No differences were observed in the OA, nor in the acrophase.

Diurnal and nocturnal QTc appeared prolonged after 60 days of HDT than after 5-day HDT (Day +3.7[1.7;6.4]%, Night +2.6[0.7;7.3]%), with a positive trend as HDT duration increases.

**FIGURE 4:**
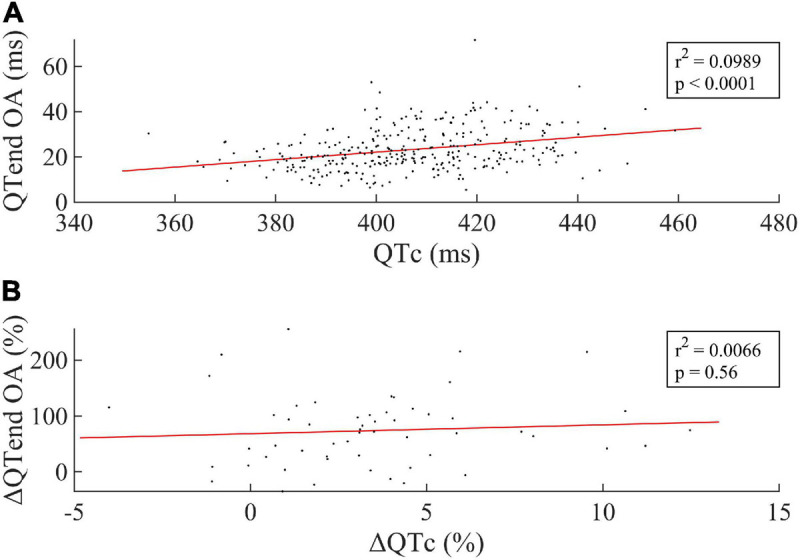
**(A)** Linear correlation between the median value of the Bazett-corrected ventricular repolarization duration (QTc) over the 24 h and the Oscillation Amplitude (OA) of the circadian rhythm in the ventricular repolarization, and **(B)** between the respective Δ(%) computed between the last HDT day and R + 0. Every dot represents one subject, while the red line represents the linear fit to the data.

Figure 4 displays the linear regression between the median QTc over the 24 h and the OA of the QTend (Figure 4A, including all acquisitions), as well as between the respective variation Δ(%) between the last HDT day and R + 0 (Figure 4B). In particular, the former showed that higher OA of the QTend were associated with longer QTc (*p* < 0.0001), while only a slight, non-significant rising trend in OA was observed with increasing QTc from the last HDT day to R + 0.

**FIGURE 5:**
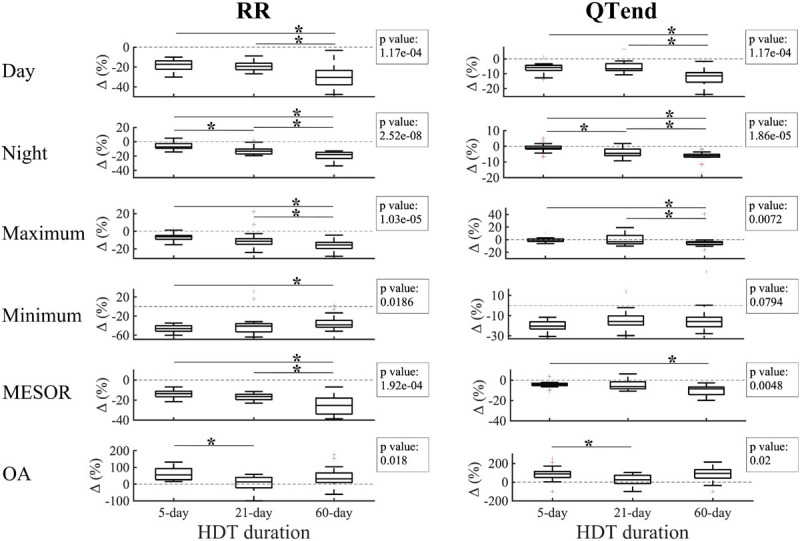
Changes (Δ %) in diurnal, nocturnal, maximum, minimum Midline Statistic of Rhythm (MESOR), and Oscillation Amplitude (OA) values for heart beat (RR, left) and ventricular repolarization (QTend, right) durations between the last day of head-down tilt (HDT) bed rest and the first day of recovery (R + 0) in 5-day, 21-day, and 60-day HDT campaigns. Dashed line indicates a null variation (Δ = 0%). In the textbox, the *p*-value of the Kruskal–Wallis test is indicated. *p < 0.05/3 *post hoc* Mann–Whitney tests with Bonferroni correction.

The effect of the deconditioning perceived at bed rest discontinuation was also studied in terms of Δ (%) between the last HDT day of 5-day (HDT5), 21-day (HDT21), and 60-day (HDT58) HDT, and the respective R + 0. As displayed in Figure 5, negative variations were found for the majority of the parameters, thus reflecting the decrease at R + 0 compared to the last day of HDT bed rest.

In particular, a larger decrease at R + 0 in the diurnal values of RR and QTend was elicited after 60-day HDT (RR: −30.5[−37.9; −23.3]%; QTend: −11.5[−15.8; −9.3]%) compared to what elicited after a 5-day HDT (RR: −17.2[−22.3; −13.8]%; QTend: −5.9[−7.7; −4.3]%) or a 21-day HDT (RR: −19.2[−22.8; −16.2]%; QTend: −6.6[−7.9; −3.2]%), while a trend with increasing HDT duration was visible during the night (RR: −7.4[−9.3; −2.6]% in 5-day, −13.1[−17.1; −10.3]% in 21-day, −18[−23.3; −14.8]% in 60-day HDT; QTend: −1.1[−1.8;0]% in 5-day, −4.5[−6.0; −1.8]% in 21-day, −6.1[−7.0; −5.1]% in 60-day HDT). Also, a trend with HDT duration was present in the maximum values, which exhibited a larger decrease after 60-day HDT (RR: −15.6[−19.6; −12.6]%; QTend: −4.2[−7.7; −3.1]%), both compared to 5-day (RR: −6.3[−9.1; −4.7]%; QTend: −1.8[−2.7;1.1]%) and 21-day HDT (RR: −11.4[−14.8; −8.4]%; QTend: −3.3[−6.4;6.8]%). As regards minimum values, no difference in QTend reduction was found, while a lower Δ was visible for RR in 60-day HDT (−38.4[−43.3; −29]%) compared to 5-day HDT (−46.3[−51.8; −40.2]%). In addition, 60-day HDT elicited a larger MESOR reduction (RR: −25.3[−34.0; −18]%; QTend: −8.5[−14.2; −6.8]%) both compared to 5-day (RR: −13.6[−16.8; −11.2]%; QTend: −4.5[−5.4; −3]%) and 21-day HDT (RR: −16.5[−19.6; −14.2]%).

As regards the OA, larger values were observed at R + 0 compared to the baseline, as evidenced by positive Δ(%). In particular, larger Δ(%) were found in 5-day HDT (RR: +55.4[26.7;93.1]%; QTend: +87.4[50.4;113.1]%) compared to 21-day HDT campaigns (RR: +13[−22;40.8]%; QTend: +24.8[−15;73.1]%).

No differences in the variation of acrophases and neither in QTc were found.

## Discussion

In this study, we retrospectively analyzed the 24 h Holter ECG acquired at different epochs during six HDT bed rest campaigns (two of 5 days, two of 21 days, and two 60 days), aiming at evaluating the effects of the HDT duration on the circadian rhythms of RR and ventricular repolarization (QT) for a large population of normal young males. To the knowledge of the authors, this study includes the widest pooled population in which the analysis of the changes induced by HDT in the circadian rhythms of cardiac electrical activity has ever been conducted.

The cumulative results confirmed our previous findings observed on data of a single campaign only (Solbiati et al., 2020), showing that the characteristics of RR and QTend circadian rhythms were affected by HDT in terms of increased RR and QTend midline and shortened QTc, reduced amplitude of day/night oscillations and slightly anticipated circadian acrophase, with larger changes visible already after the first 5 days of HDT bed rest. The conclusion of the HDT elicited opposite changes, including the shortening of RR and QTend, and the prolongation of the QTc, as well as the increased OA and a slightly delayed acrophase during the recovery stage. In addition, we were able to evaluate the degree of positive dependence of post-HDT changes with increasing HDT duration.

### Effects on Intervals Duration

Within the first 5 days of HDT, the midline value (MESOR) of RR and QTend intervals over the 24 h recording increased, mostly due to the substantial lengthening of the RR interval during the day and to the increased minimum values with unaffected maxima. These results are in accordance with the study of Liang et al. (2014) on eight healthy men performing a 45-day head-down bed rest, evidencing that the heart rate decrease over the 24 h mainly occurred at the beginning of the HDT. The detected tendency toward bradycardia well reflects what observed in short-duration spaceflights (Fritsch-Yelle et al., 1996; Beckers et al., 2003) and is mainly attributable to the physiological adaptation to the new condition of tilted, bedridden immobilization, including circulatory unloading and decreased daily activity. In turn, this resulted in a prolongation of the QTend interval, while the QTc resulted shortened, suggesting the existence of an effect of HDT on the repolarization phase.

Afterward, a progressive trend toward baseline values was observed, with the QTend recovered already at HDT21, and RR at HDT58. A similar progressive recovery was also described in other bed rest studies, with heart rate resulting even increased after 35–45 days of bed rest (Pavy-Le Traon et al., 2007; Liang et al., 2014; Liu et al., 2015). In particular, Liang et al. (2014) concluded that this readjustment could be attributed to the chronic head-down position, since it occurred under constant level of activity within the bed rest, recorded by a wrist accelerometer. On the contrary, results from long duration spaceflights reported no changes in inflight heart rate obtained from Holter ECG recordings both during daily activities (Fraser et al., 2012) and during sleep (Xu et al., 2013), from early (2–4 weeks after launch) to later in-flight (2–4 weeks before landing) acquisitions compared to pre-flight, as well as in resting heart rate extracted from 10-min recordings (Baevsky et al., 2007). Additionally, Yamamoto et al. (2015) found that the mean RR interval from 24-h Holter ECG, after an initial increase observed in 5 out of 7 astronauts during ISS expeditions 1 month after launch, progressively modified in a subject-specific way, with three astronauts developing high bradycardia, two with mild bradycardia, and two with tachycardia.

The subsequent restoration of the orthostatic position elicited opposite variations, independently from the HDT duration, with abrupt RR and QTend shortening when the normal level of gravity on the head-to-foot axis and body fluid distribution were restored, confirming what observed after a period of permanence under real (Beckers et al., 2003) or simulated (Liang et al., 2014) microgravity. Results from 5-day HDT campaigns indicate that even a short exposure to a simulated microgravity environment is enough for eliciting the deconditioning of the cardiac circadian timing system, resulting in the inability to compensate for the re-established gravitational condition, as highlighted by the increased RR and QTend intervals still 4/5 days after the HDT conclusion. Similarly, 5/6 days and 6–8 days after HDT termination, respectively in 21-day and 60-day, were not sufficient for restoring the circadianity of RR and QTend intervals to baseline values. Indeed, data from short duration (10–14 days) space flights revealed that a period between 5 (Beckers et al., 2009) and 25 days (Verheyden et al., 2007) is needed for a complete recovery and, similarly, maximum and minimum heart rate values remained increased also 10–12 days after a 45-day head-down bed rest (Liang et al., 2014).

Interestingly, the shortening of RR and QTend intervals was visible also during the night period, when the subjects were in horizontal position as before the HDT, similarly to what observed after long-duration spaceflight (Xu et al., 2013): this suggests a bed rest-induced impairment of the autonomic control system, confirmed by long-duration spaceflight studies (Fritsch-Yelle et al., 1994; Baevsky et al., 2007), possibly maintaining altered RR and ventricular repolarization intervals also at night, when the head-to-foot gravity load is removed. In addition, similar and simultaneous variations in RR and QTend were observed, indicating that the relation between RR and cardiac repolarization is maintained.

Additionally, the magnitude of post-HDT changes appeared to increase with prolonged HDT duration, with the differences between the last day of the HDT and R + 0 being significant in 5-day, 21-day and, even more markedly, in 60-day HDT campaigns. As circadian desynchronization could be related to a decrease in astronaut’s performance, this observation can be relevant for future manned space exploration scenarios, including the return to the Moon and missions to Mars, where the return to a gravitational field (hence reduced compared to the Earth) after a period (for Mars, at least 9 months) of permanence in weightlessness could generate abrupt perturbation of the cardiac electrical activity circadianity in respect to the previous homeostasis reached during space flight. This could also be put in relation with the increased electrical instability, reflected by the increase in T-wave alternans indices computed on the 24-h, after 60-day exposure to bed rest that we found in a previous study (Martín-Yebra et al., 2019) conducted on the same pooled population, thus revealing that ventricular repolarization mechanisms may also be altered during this period.

Moreover, the QTc resulted significantly increased after the HDT, particularly after 21-day and 60-day HDT, when the maxima exceeded the 450 ms physiologic limit defined by the AHA/ACCF/HRS Consensus Document (Rautaharju et al., 2009) for long QT. Similarly, other studies have observed a prolongation of the QTc during long duration spaceflight (D’Aunno et al., 2003; Golubchikova et al., 2003), while no prolongation was reported in short duration spaceflight (Mitchell and Meck, 2004). The prolongation of the Bazett-corrected QT interval has been related to increased risk of developing torsade de pointes, a life-threatening polymorphic ventricular tachycardia, possibly degenerating into ventricular fibrillation (Kallergis et al., 2012). Generally, the non-hereditary QT prolongation is observed with the exposure to an environmental stressor that, in the case of HDT studies or spaceflight, could corresponds to the restoration of Earth-like, or partial (Moon or Mars) orthostatic gravitational stimuli after a period of permanence in microgravity, respectively.

Normally, prompt intervention could be life-saving in case of adverse cardiac electrical event. However, in the scenario of a return to the Moon, an emergency re-entry to the Earth or to a low Earth orbit base (if any) could take days, and would be unfeasible in missions to Mars. For this reason, the development of effective countermeasures for preventing the spaceflight-induced cardiovascular deconditioning will be paramount.

### Effects on the Circadian Amplitude

An important feature of circadianity in a time series is represented by the amplitude of day-night fluctuations. It was previously observed that the amplitude of 24-h heart rate significantly reduced during a 45-day HDT bed rest (Liang et al., 2012), both compared to baseline and to post-HDT, as well as during a 60-day HDT bed rest (Solbiati et al., 2020).

In this study we confirmed a significant reduction in the oscillation amplitude (OA) of both RR and QTend circadian rhythms in both early (5 days), mid (21 days), and late (58 days) HDT. The reduced physical activity/rest cycle and the chronic elimination of the upright/supine postural cycle, both important environmental synchronizers, may have contributed to the observed loss in physiological RR and QTend day-night oscillation during the HDT. Interestingly, this reduction was already present after only 5 days of immobilization, and kept stable with the continuation of HDT bed rest. A reduction in the circadian amplitude may additionally underline an autonomic disfunction. Also, it is associated to a decreased capacity of adaptation of the physiological system to the presentation of new external stimuli, which manifested as increased QTend OA immediately after 5-day and 60-day HDT, a condition that has been associated to increased risk of arrhythmic events in cardiac patients (Du Pre et al., 2017). A recent study (Du Pre et al., 2017) showed that the day-night QT oscillation amplitude was higher in patients with confirmed or potential (Solatol-induced) QT prolongation. Similarly our results showed that the observed rise in the oscillation amplitude at R + 0 was concurrent with the prolongation of the QTc.

### Effects on the Circadian Acrophase

In this study, a backward shift of the RR and QTend acrophase was visible particularly at early (5th day) and late HDT (57th–58th day), while it was postponed at R + 0 after both 21-day and 60-day HDT, similarly to what observed by Liang et al. (2014) in a 45-day HDT bed rest campaign. Interestingly, the described variations occurred under controlled and fixed sleep-wake and feeding schedule, thus underlying that the reduced physical activity and circulatory unloading may have a role in the temporal entrainment of cardiac circadian rhythms.

The reduced daily physical activity and the elimination of the upright/supine postural diurnal changes contributed to the acrophase alterations, while the preservation of controlled lighting and feeding times possibly helped maintaining the rhythm entrained around the 24 h. However, during spaceflight, the absence of the 24-h light/dark cycle, which is the major synchronizer, could lead toward a free-run, possibly disrupting the physiological circadian rhythms. A desynchronization of the circadian rhythm is a risk factor for chronic diseases such as metabolic syndromes, diabetes, hypertension, obesity, cardiovascular disease, cancer, behavioral disorders, impaired hormonal, endocrine, immune and autonomic functions (Buijs et al., 2016), and critical outcomes have been observed also in long-term shift workers (Haus and Smolensky, 2013). Additionally, the difference (Δφ) between φ_*QTend*_ and φ_*RR*_ reduced at HDT5, as well as at R + 5/7 after 60 days of HDT, thus indicating that the phase of RR and QTend circadian cycle coupling may be also affected by microgravity.

### Limitations

The Bazett formula is currently the most widely QT correction method used in clinical standards (Vandenberk et al., 2016). However, the utilization of Bazett correction may not be appropriate, as it is known to overcorrect the QT interval at fast heart rates, such as during the recovery phase, and to undercorrect at lower heart rates, as within the first days of bed rest (Malik, 2001).

In the present study, the availability of only 24-h ECG recordings duration prevented the application of other non-linear, frequency or time-frequency domain analysis methods (i.e., requiring availability of multi-days recording) to investigate possible changes induced in the circadian period.

As the respiratory activity was not directly monitored during the experiments, baroreflex interactions, cardio-respiratory coupling and synchronization were not taken into account and were beyond the aim of this study. Alterations in the baroreflex sensitivity, governing the cardio-respiratory coupling through respiratory sinus arrhythmia, were also observed in chronic exposure to HDT bed rest (Iwasaki et al., 2000, 2004). As well, respiratory frequency was found decreasing during long-term spaceflight and considerably increasing after landing, as observed by 10-min acquisitions (Baevsky et al., 2007). Accordingly, further studies could integrate this investigation by extracting the respiratory frequency indirectly from the ECG (Helfenbein et al., 2014), thus providing additional insights to the deconditioning of autonomic modulation and cardio-respiratory interaction elicited by microgravity.

## Conclusion

In this study, a retrospective analysis on the changes in the circadian rhythms of RR and ventricular repolarization intervals induced by head-down bed rest was conducted. A particular strength of this work is the availability of a large number of 24-h Holter ECG recordings acquired during six past HDT campaigns from a pooled group of normal subjects (*n* = 63 in the non-intervention group), which is, to the knowledge of the authors, the widest population ever studied in related literature.

The first aim was to evaluate the degree and the progression of circadian desynchronization from 5 to 60 days of HDT. Major changes were observed within the first days of the HDT, with increased RR and QTend intervals and decreased QTc. Afterward, a progressive trend toward baseline values was observed: the recovery was reached already at HDT21, except for the RR and QTend minima and the QTc maxima. Conversely, the amplitude of day-night oscillations resulted significantly decreased from acute to chronic HDT, showing no tendency to recover, and the acrophase anticipated.

The restoration of the normal head-to-foot gravity field elicited opposite variations, including the shortening of RR and QTend intervals and QTc prolongation, notwithstanding duration of the period spend in HDT. However, the entity of the changes observed at R + 0, as well as the perceived deconditioning expressed as difference between the last HDT day and R + 0, showed a trend of dependence with HDT duration, where longer HDT producing major deconditioning at gravity restoration, and 5–7 days being not sufficient for recovering after 60-day HDT. Interestingly, the increased OA at R + 0 after 5-day and 60-day HDT suggests impaired ability to compensate for the re-established gravitational condition as a consequence of the HDT, with possible involvement of the autonomic control of cardiac activity, and possibly leading to increased arrhythmic risk.

These results provide evidence of the importance of monitoring astronaut’s circadian rhythms, as an impaired circadian timing system could generate adverse health outcomes and decreased performance. The investigation of measures aimed at counteracting the observed modifications would be paramount for preserving astronauts’ health and mission success in manned space exploration, including the return to the Moon and missions to Mars.

## Data Availability Statement

The datasets analyzed in this article are not publicly available due to constraints relevant to EU General Data Protection Regulation. Requests to access the datasets should be directed to European Space Agency.

## Ethics Statement

The studies involving human participants were reviewed and approved by Institutional Review Board of the “Comité de Protection des Personnes Sud Ouest et Outre Mer I” and by the Institutional Review Board of the Deutsches Zentrum für Luft- und Raumfahrt (DLR). The patients/participants provided their written informed consent to participate in this study.

## Author Contributions

EC and PV conceived and designed the research. EC and AM-Y contributed to the data acquisition. SS and AM-Y contributed to the development of analytical tools. SS analyzed the data. SS and EC wrote the manuscript. All the authors read and approved the manuscript.

## Conflict of Interest

The authors declare that the research was conducted in the absence of any commercial or financial relationships that could be construed as a potential conflict of interest.
