# Supplementary material for: Evaluation of Cardiac Circadian Rhythm Deconditioning Induced by 5-to-60 Days of Head-Down Bed Rest
Source: Front Physiol. 2021 Jan 13;11:612188. doi: 10.3389/fphys.2020.612188 (PMC7838678; doi:10.3389/fphys.2020.612188)
Supplement: Supplementary file 1 [file Table_1.docx]

Supplementary Material

In Supplementary Table 1, the details for each campaign relevant to its European Space Agency official acronym, when and in which bed rest facility it was conducted, the total number of subjects enrolled and the number of those that successfully terminated the experiment when assigned in the no-intervention (CTRL) group, their age range, and the duration of each phase (PRE, HDT, recovery), are reported.

**Supplementary Table 1.** Summary of details relevant to the head-down tilt (HDT) bed rest campaigns conducted at the Institut de Médecine et de Physiologie Spatiales (MEDES), or at the German Aerospace Center (Deutsches Zentrum für Luft- und Raumfahrt e.V, DLR), with total enrolled male subjects and number of subjects assigned to the non-intervention group (CTRL) that completed the experiment, from which 24h Holter ECG was acquired. PRE: days of observation before HDT was started; Recovery: days of observation after HDT was terminated.

| HDT campaign acronym | Year | Place | Total Subjects | Subjects in CTRL group | Age range  (years) | PRE  (days) | HDT  (days) | Recovery  (days) |
| --- | --- | --- | --- | --- | --- | --- | --- | --- |
| BR-AG1 | 2010 | MEDES | 12 | 12 | 21÷41 | 5 | 5 | 5 |
| SAG | 2010/  2011 | DLR | 10 | 10 | 25÷44 | 5 | 5 | 5 |
| MEP | 2011/  2012 | DLR | 10 | 9/10 | 23÷42 | 7 | 21 | 6 |
| MNX | 2012/  2013 | MEDES | 12 | 11/12 | 20÷44 | 7 | 21 | 6 |
| RSL | 2015/  2016 | DLR | 24 | 11/12 | 20÷45 | 14 | 60 | 14 |
| Cocktail | 2017/  2018 | MEDES | 20 | 10 | 20÷45 | 14 | 60 | 14 |

# 5-day HDT best rest

# The two 5-day HDT campaigns (BR-AG1 and SAG) were designed as randomized cross-over studies, with every subject repeating the HDT bed rest three times (washout period of 1.5 months in-between), one time while in the control group (CTRL) and two times in a specific countermeasure (CM) group applied during HDT, in random order among the three campaigns.

# A period of acclimatization of 5 days inside the bed rest facility for baseline data collection, defined as PRE, preceded the 5-day HDT, as well as a period of 5 days post-HDT recovery, defined as R, followed the termination of the HDT, starting in the morning of the 6^th^ day (R+0).

For each repetition in each campaign, the Holter-24h experiment was started the day the subject arrived in the bed rest facility to obtain baseline values, the fifth day of HDT (HDT5) to measure the whole effects induced by bed rest, the following day (R+0) to observe immediate changes of returning to normal alternation of horizontal-vertical position, and four (R+3, in BR-AG1) or five (R+4, in SAG) days after, to study the process of restoration to baseline values.

# 21-day HDT best rest

# The two 21-day HDT campaigns were designed as randomized cross-over studies, with every subject repeating the HDT bed rest two (MEP) or three (MNX) times (washout period of 4 months in-between), one time while in the control group (CTRL) and one or two times in a specific countermeasure (CM) group applied during HDT, in random order among the campaigns.

# A period of acclimatization of 7 days inside the bed rest facility for baseline data collection, defined as PRE, preceded the 21-day HDT, as well as a period of 6 days post-HDT recovery, defined as R, followed the termination of the HDT, starting in the morning of the 22^th^ day (R+0).

For each repetition in each campaign, the Holter-24h experiment was started the day the subject arrived in the bed rest facility (MEP) or two days later (MNX) to obtain baseline values, the fifth day of HDT (HDT5) to assess early changes, the 21^th^ day (HDT21) to measure the whole effects induced by bed rest, the following day (R+0) to observe immediate changes of returning to normal alternation of horizontal-vertical position, and five (R+4, in MEP) or six (R+5, in MNX) days after, to study the process of restoration to baseline values.

# 60-day HDT best rest

# The two 60-day HDT campaigns (RLS and Cocktail) were designed as multi-group, with every subject performing only one session and being randomly assigned either to the CTRL or to the CM group.

# A period of acclimatization of 14 days inside the bed rest facility for baseline data collection, defined as PRE, preceded the 60-day HDT, as well as a period of 14 days post-HDT recovery, defined as R, followed the termination of the HDT, starting in the morning of the 61^th^ day (R+0).

For each campaign, the Holter-24h experiment was started five (RLS) or six (Cocktail) days after the subject arrived in the bed rest facility to obtain baseline values, the fifth day of HDT (HDT5) to assess early changes, the 21^th^ day (HDT21) to evaluate mid-term changes, and the 57^th^ (RLS) or the 58^th^ day (Cocktail) to measure the late effects induced by bed rest, the following day (R+0) to observe immediate changes of returning to normal alternation of horizontal-vertical position, and six (R+5, in RLS) or eight (R+7, in Cocktail) days after, to study the process of restoration to baseline values.
